# Early loss of axons limits the therapeutic window in a mouse model of spinal muscular atrophy

**DOI:** 10.1186/s12868-026-01034-1

**Published:** 2026-08-06

**Authors:** Laura H. Comley, Inga Partlova, Charles Nyasa, A’ishah Shaikh, Lyndsay M. Murray

**Affiliations:** 1 Institute for Neuroscience and Cardiovascular Research, University of Edinburgh, Edinburgh, EH8 9XD UK; 2 Euan MacDonald Centre for Motor Neuron Disease Research, University of Edinburgh, Edinburgh, EH16 4SB UK; 3 College of Medicine and Veterinary Medicine, University of Edinburgh, Hugh Robson Building, George Square, Edinburgh, EH8 9XD UK

**Keywords:** Motor neurons, Spinal muscular atrophy, Axon, Mouse models, SMN-upregulating therapy

## Abstract

Current treatments for spinal muscular atrophy have significantly improved patient prognosis, but still fall short of a full cure, particularly when administered after symptom onset. To improve future therapies, it is essential to identify the early pathological events that currently limit treatment efficacy. Here we have investigated the vulnerability and maturation of intramuscular motor axons in the SmnΔ7 mouse model of spinal muscular atrophy using a panel of cranial muscles with differing susceptibility to disease. We demonstrate that although there is intermuscular heterogeneity in neuromuscular junction pathology, they are united by a common loss of motor axons. We show that the timing of motor axon loss differs between muscles, but that motor axon loss precedes structural denervation and can occur by postnatal day 1 in highly vulnerable muscles. Electron microscopy of intramuscular axons revealed that axonal maturation and myelination are grossly normal prior to subsequent postnatal degeneration. We note that axons lack the traditional morphological correlates of axon degeneration but can be visualised using antibodies which detect degenerating neurofilaments. Treatment with SMN-upregulating therapy SMN-C8 at best preserves intramuscular axons when administered before the onset of degeneration but is unable to reverse damage or promote regeneration once axon loss has occurred. Collectively this data demonstrates that SMN-upregulating therapy can prevent motor axon loss, but the capacity is limited by the number of intramuscular axons which remain at the time of treatment onset. This work highlights the importance of developing complementary therapies which can promote motor axon regeneration to act in synergy with SMN-upregulating compounds.

**Supplementary Information:**

The online version contains supplementary material available at https://doi.org/10.1186/s12868-026-01034-1.

## Introduction

Spinal muscular atrophy (SMA) is a genetic disorder characterised by the progressive loss of motor neurons, leading to muscle atrophy and paralysis and is caused by mutations in the *SMN1* gene [1]. Treatments for spinal muscular atrophy have advanced significantly in recent years, focusing on gene replacement therapy of the *SMN1* gene (onasemnogene abeparvovec [2]), and *SMN2*-modifying therapies (nusinersen [3] and risdiplam [4]), all of which increase the level of functional SMN protein [5]. However, while current treatments for SMA have dramatically improved patient prognosis, they remain therapies rather than cures. Outcomes vary widely, and many people with SMA who receive treatment still fail to achieve motor milestones and/or have skeletal deformities, persistent fatigue, pain and the need for ongoing nutritional and respiratory support [6–9]. Even infants treated during the clinically silent phase within the first six weeks of life following newborn screening can still exhibit impactful motor deficits [10–12]. It is therefore critical to understand what is limiting treatment efficacy if we are to optimise the next generation of therapies.

SMA is characterised by a progressive loss of motor neurons that leads to profound muscle weakness and atrophy. Motor neuron breakdown occurs in a distal to proximal pattern beginning at the neuromuscular junction (NMJ) [13–15]. Within this synapse, impaired calcium signaling [16] and reduced synaptic vesicle release [17] lead to perturbed neurotransmission, causing functional deficits in muscle contraction. This is followed by denervation of the NMJ and loss of the cell body. Importantly, not all muscles are equally affected in SMA, some muscles have high levels of denervation while others remain intact late in disease [13, 18, 19]. The reasons for this selective vulnerability remain unknown although a number of important modifiers have been identified [20–23].

SMN is expressed at high levels during embryogenesis [24], suggesting it could play a role in prenatal development and raising the possibility of problems with neurodevelopment. Developmentally immature motor neuron axons are present in the ventral roots of both SMA patients and mouse models [25]. These axons have impaired radial growth and reduced ensheathment by myelin and degenerated rapidly perinatally [25]. It is unclear whether these immature axons observed in the ventral roots of SMA patients and mice are functionally connected to muscle and/or have the capacity to become functionally connected following treatment.

With the advent of treatments for SMA, it is important to consider how the motor unit responds to an increase in functional SMN levels and the extent to which it can recover. We have previously undertaken a study in the SmnΔ7 mouse model of SMA using an experimental antisense oligonucleotide (ASO), examining how the motor unit responds to treatment [26]. This revealed that following early treatment, remaining motor endplates were rapidly reinnervated. However, there was a persistent reduction in the number of intramuscular motor axons with an associated increase in average motor unit size. These defects spread to less vulnerable muscles when treatment was delayed by a few days [26]. We have further shown that the decrease in axon number persists following dual administration of Nusinersen and SMN-C8, despite a significant enhancement in Smn levels (Partlova et al., In Press). Significant gaps remain in our understanding of the neuromuscular system in SMA and its response to treatment. Specifically, does motor axon regeneration occur following treatment? How does the stage of the disease and the relative vulnerability of the muscle affect the capacity for regeneration and/or axon preservation? To answer these, it is important to understand the extent of motor axon loss from intramuscular bundles at the time of treatment; how axonal loss manifests over time and the extent to which axon numbers stabilise, regenerate or continue to decline following treatment.

To address these knowledge gaps, we have investigated motor axon vulnerability in a panel of cranial muscles with differing susceptibility to disease in the SmnΔ7 mouse model of SMA [27]. Here we show that intramuscular motor axon loss occurs early in SMA, preceding denervation of the neuromuscular junction. Vulnerable motor axons in the cranial muscles of SmnΔ7 mice exhibit grossly normal maturation and myelination before undergoing subsequent degeneration, suggesting that the postnatal degenerative process of these axons is not primarily driven by defects in axonal development or myelination. We further show that axons do not exhibit classical signs of degeneration, and instead become negative for traditional labelling of neurofilaments while the cytoskeleton degenerates. SMN-upregulating therapy (SMN-C8) can at best preserve existing axons when administered before degeneration begins, but it is unable to reverse prior axonal loss, highlighting a critical window for treatment efficacy. Ultimately, these findings demonstrate that intramuscular motor axon degeneration is a primary, early pathological event in SMA which cannot be reversed by SMN-upregulation.

## Materials and methods

### Mouse breeding and treatment

Smn+/-;SMN2+/+;SMNΔ7+/+ mice (Jackson strain 005025, referred to as SmnΔ7 mice, FVB congenic background) were maintained in breeding pairs at the animal facilities at the University of Edinburgh. This cross produced experimental Smn-/-;SMN2+/+;SMNΔ7+/+ pups and heterozygous and homozygous control littermates. For clarity we refer to Smn-/-;SMN2+/+;SMNΔ7+/+pups as ‘SmnΔ7’ throughout, and Smn+/-;SMN2+/+;SMNΔ7+/+ or Smn+/+;SMN2+/+;SMNΔ7+/+ mice as ‘controls’. All genotyping was performed using standard PCR protocols on DNA extracted from tail tips (taken post-mortem). Mice over postnatal day 5 (P5) were sacrificed by overdose of inhalation anaesthetic isoflurane, and death was confirmed by exsanguination from the carotid artery. Mice under P5 were decapitated under terminal anaesthesia and death was confirmed by exsanguination from the carotid artery.

For SMN-upregulating treatment mice pups received a daily dose of SMN-C8 (RO7021707, given by generous agreement with Roche). SMN-C8 was prepared in a solution of 20% Kleptose^®^ / 10 mM lactic acid in water, with final pH adjusted to 5.8, and stored at -20 °C. SMN-C8 was administered at a dose of 1 mg/kg via intraperitoneal injection starting at either P2 or P3, where day of birth was considered P0. Pups were monitored following all procedures, and daily weights, righting reflex test and hind-limb splay motor tests were performed. All experiments were performed in accordance with the regulations set out by the UK Home Office.

### Immunohistochemistry

Cranial muscles from the back of the head and neck were immediately dissected from sacrificed mice and fixed in 4% paraformaldehyde (Electron Microscopy Science) in PBS for 15 min. Secondary dissection was then used to isolate individual cranial muscles; levator auris longus (caudal: LALc), levator auris longus (rostral: LALr), auricularis superior (AS) and abductor auris longus (AAL). All muscles were permeabilised in 2% Triton X-100 in PBS for 30 min, before incubation overnight in primary antibodies against neurofilament medium (to capture axons at early postnatal stages) and synaptic vesicle proteins (NF-M (2H3), 1:50; SV2, 1:100; Developmental Studies Hybridoma Bank) and a further four-hour incubation with secondary antibodies (AlexaFluor-488 rabbit anti-mouse at 1:250; Jackson). Post-synaptic acetylcholine receptors (AChRs) were labelled with α-bungarotoxin (BTX) (1:250; tetramethylrhodimine conjugate; Life Technologies) for 30 min. Muscles were then whole mounted in Mowiol (Sigma).

For co-staining with neurofilament-medium (NF-M) and degenerated neurofilament-light (NF-L DG-Sensor™, 1:200; 2B scientific), muscles were initially incubated overnight in primary antibodies against DG-Sensor [28], followed by a four-hour incubation with secondary antibodies (AlexaFluor-568 goat anti-chicken at 1:250, Jackson), before being incubated overnight with primary antibodies against NF-M and a second four hour incubation with secondary antibodies (AlexaFluor-488 rabbit anti-mouse at 1:250, Jackson). Muscles were then mounted as previously described.

### Quantification and analysis

All quantification was performed with researcher blind to genotype and treatment group.

#### Innervation

Innervation of the NMJ was determined by analysing a minimum of 50 endplates across the entire endplate band of a muscle. Each endplate within a field of view was categorised as either fully occupied (the presynaptic terminal completely overlies the endplate), partially occupied (the presynaptic terminal partially covers the endplate) or vacant (no presynaptic staining overlies the endplate).

#### Axon number

Axon number was counted in a consistent anatomical region of each muscle (detailed Supplementary Fig. 1A and B). Three counts were made and the highest was used for analysis, to minimise underestimating axon number due to technical limitations such as overlapping fibres obscuring individual axons in specific focal planes. Accuracy of these counts was confirmed using confocal Z-stacks which could be rotated to allow for visualisation of the cross section of an intramuscular axon bundle (Supplementary Fig. 1C).

#### Motor unit size

Average motor unit size was calculated by dividing the total number of endplates with any axonal contact (both fully occupied and partially occupied) by the total number of axons in the intramuscular bundle.

#### Presynaptic swelling

For presynaptic swelling, all NMJs were classified as having no swelling, mild (evidence of some axonal swelling and/or presynaptic swelling that does not obscure the end plate), moderate (evidence of clear axonal swelling and significant presynaptic swelling that is beginning to obscure the end plate) or severe (significant and obvious swelling along the length of the axon and severe swelling around the presynaptic terminal, which obscures the endplate).

#### Endplate area and number

Total endplate number was counted using an inverted Leica microscope, on entire whole mount muscles. Endplate number was assessed by counting the number of acetylcholine receptor (AChR) clusters within an anatomically consistent area of the muscle (LALc, C2; LALr, R3; AS and AAL, entire medial endplate band [13, 29]). Endplate size was quantified using ImageJ software. At least three fields of view totalling > 50 endplates per muscle were analysed.

### Electron microscopy

Cranial muscles from the back of the head and neck were immediately dissected from recently sacrificed mice and fixed in EM fixative (4% paraformaldehyde, 2.5% glutaraldehyde in 0.1 M phosphate buffer, pH 7.4) overnight. Intramuscular endplate bundles were then dissected from LALc and LALr muscles.

Muscle samples were washed 3 times with 0.1 M phosphate buffer (pH 7.4; Sigma) then incubated in 1% v/v osmium tetroxide (TAAB) in 0.1 M phosphate buffer for 30 min, before 3 further washes in phosphate buffer. Samples were then dehydrated through an ascending ethanol series (50%, 70% [including 1% w/v uranyl acetate], 95%, and 100%) and acetone (Electron Microscopy Services). After dehydration, the samples were lifted into Durcupan resin (Sigma) and left overnight at room temperature. Next day, the samples were lifted into fresh resin in molds and cured at 65 °C over three days.

Resin-embedded samples were sectioned using a Leica Ultracut UC7 ultramicrotome and 70 nm-thick sections collected onto Formvar/carbon-coated single-slot copper grids (TAAB). These were then contrasted with uranyl acetate and lead citrate (Sigma) before imaging.

Entire intramuscular axon bundles were imaged at 3-6000x magnification using a Zeiss Gemini 360 scanning electron microscope (SEM) with a 12-grid carousel sample holder and a dedicated scanning transmission EM (STEM) detector. Axons were assigned a maturation stage based on morphology and relationship with a surrounding Schwann cell and pseudo coloured for ease of quantification in Photoshop. Axon area and the outer area of the myelin sheath plus axon were measured within ImageJ software and used to calculate G-ratio.

## Results

### Profiling of neuromuscular pathology in cranial muscles highlights early axonal loss in SmnΔ7 mice

**Fig. 1:**
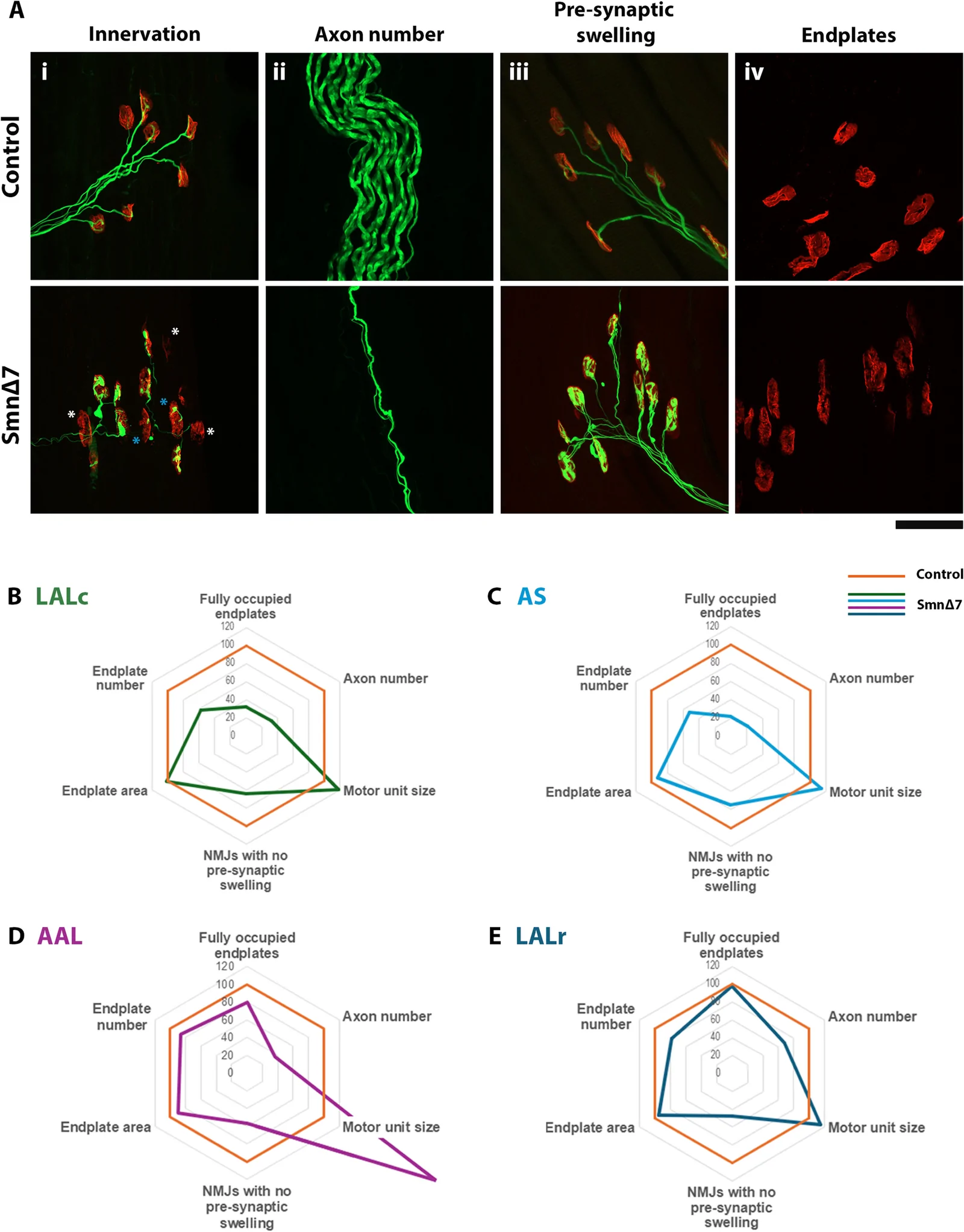
Profiling of neuromuscular pathology in cranial muscles highlights early axonal loss in SmnΔ7 mice (**A**) Confocal micrographs of pathology in cranial muscles of control and SmnΔ7 mice at end stage of disease (P12). Axons are shown in green (labelled with antibodies against neurofilament medium (NF-M) and synaptic vesicle protein 2 (SV2)) and motor endplates on muscle fibres are shown in red (bungarotoxin marking acetylcholine receptors). (**i**) Endplates in healthy control muscles are all innervated by incoming axons which form nerve terminals overlying the endplates. In SmnΔ7 muscles some endplates are vacant (white asterisks) or only partially innervated by incoming axons (blue asterisks), evidencing degeneration in these muscles. (**ii**) Incoming intramuscular axon bundle innervating the LALc muscle in control and SmnΔ7 mice, showing the massive loss of axons in SmnΔ7. (**iii**) Nerve terminals in SmnΔ7 muscle showing evidence of presynaptic swelling, compared to control muscle where nerve terminals overlying endplates are comprised of fine terminal arborisation. (**iv**) Endplates in control and SmnΔ7 muscles. Scale bar = 50 μm. (**B-E**) Radar plots showing quantification of multiple parameters of NMJ and intramuscular axon pathology in four differentially vulnerable cranial muscles. Orange hexagons represent control data for each muscle, coloured lines show data from SmnΔ7 mice, normalised to control (*n* = 3 mice)

Previous work has highlighted the early loss of NMJs in mouse models of SMA. However, we have also shown that whilst muscle innervation can recover following treatment there is frequently a persistent loss of motor axons and endplates in vulnerable muscles [26]. It is therefore important to address the temporal breakdown of the motor unit in differentially vulnerable muscles. In this study we have used a panel of cranial muscles with differing susceptibility to disease in the SmnΔ7 mouse model of SMA (Supplementary Fig. 1). These are muscles of facial expression which are responsible for ear movement in mice and, due to their small and thin dimensions, can be whole mounted flat, allowing quantification of a range of structural parameters of the motor unit. We assessed six parameters of NMJ / distal motor unit pathology in these muscles: endplate innervation, axon number, average motor unit size, pre-synaptic swelling, endplate area and endplate number (Fig. 1).

Quantification of all parameters in four cranial muscles in late stage (P12) control and SmnΔ7 mice can be visualised using radar plots where an equilateral hexagon reflects the normal healthy control values and the extent of deviation from this shape reflects the relative pathology within the muscle (Fig. 1B-E). This analysis reveals significant intermuscular heterogeneity in levels of neuromuscular pathology. The LALr displays the least deviation from the healthy control, with normal levels of innervation, endplate area and motor unit size (Fig. 1E). Despite the apparent resistant status of this muscle, there is modest but significant concurrent loss of endplates and axons, and presynaptic swelling. However, the profile of LALr deviates dramatically from the AS and LALc muscles (Fig. 1B and C). LALc and AS display a remarkably similar profile, with high levels of denervation, loss of axons, loss of endplates and modest pre-synaptic swelling. Notably, the concurrent loss of axons and endplates and high levels of denervation result in no change to the average motor unit size. The AAL reveals a unique profile where a dramatic loss of axons occurs in the absence of denervation or endplate loss, resulting in a significant increase in the size of the motor unit (Fig. 1D). This indicates that the AAL muscle possesses some intrinsic plasticity which can compensate for ongoing degeneration. These distinct muscle profiles underline the heterogeneity that exists between muscles and the importance of considering multiple parameters and multiple muscles when evaluating neuromuscular pathology. Notably, the most consistent feature across muscles is a reduction in motor axon numbers across muscles with varying disease susceptibility. This prompted further investigation into the timing of axon loss and the potential for rescue through current SMA therapies.

### Loss of axons precedes structural denervation in SmnΔ7 mice

**Fig. 2:**
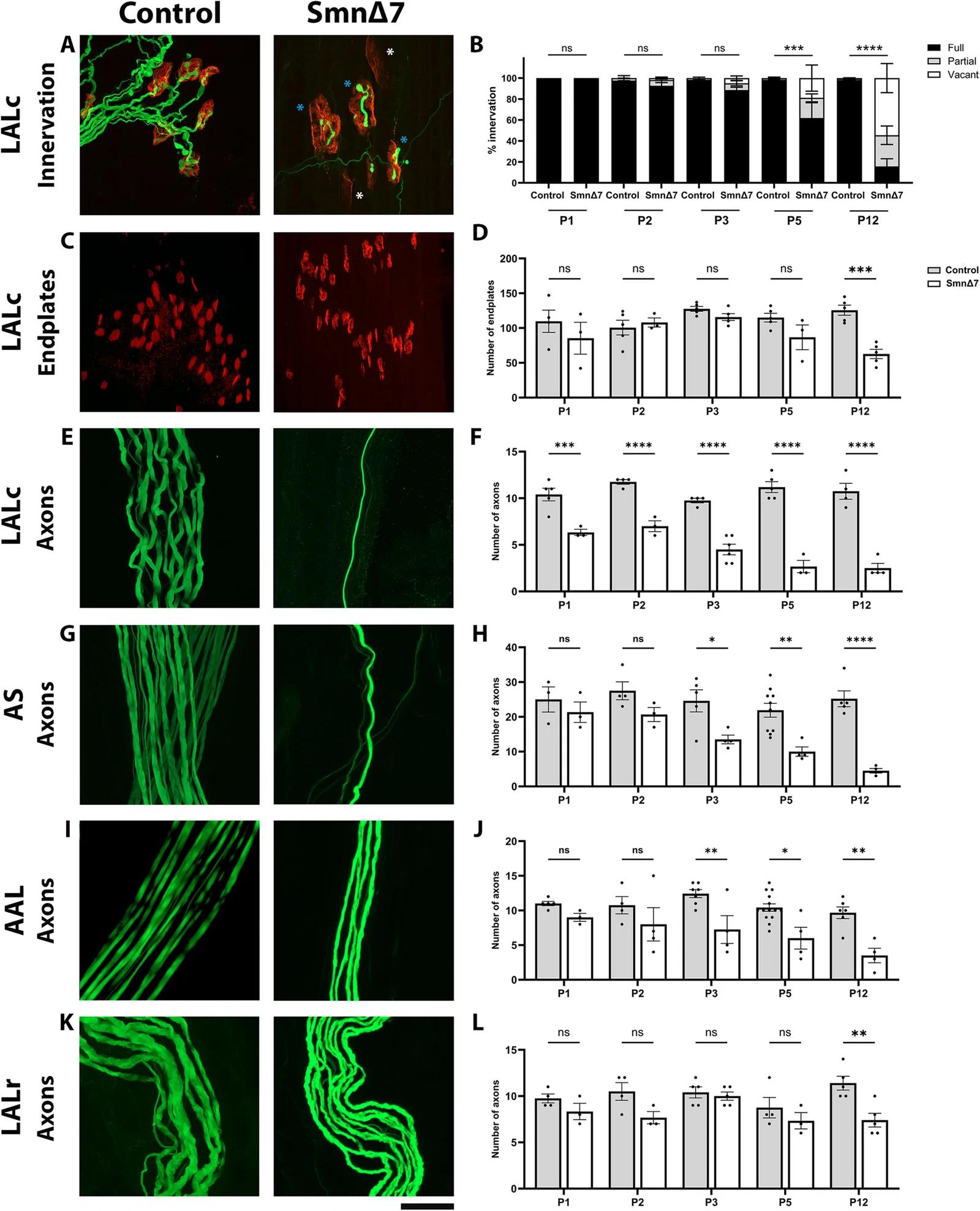
Loss of axons precedes structural denervation in SmnΔ7 mice (**A**) Confocal micrographs showing fully innervated endplates in a control LALc muscle compared to vacant (white asterisks) and partially innervated endplates (blue asterisks) in an SmnΔ7 littermate at end stage (P12). (**B**) Bar chart showing fully innervated endplates (black), partially innervated endplates (grey) and vacant endplates (white) in control and SmnΔ7 LALc muscles across time. There was a significant loss of innervation in the LALc by 5 days of age. (**C**) Micrographs showing part of the endplate band in the LALc of control and SmnΔ7 mice. (**D**) Bar chart showing total number of endplates in the LALc in control (grey) and SmnΔ7 mice (white) across time, from P1 to P12. There was a 50% reduction in endplate number in the SMA muscles by P12. (**E**) Micrographs showing axons innervating the LALc muscle in control and SmnΔ7 mice at end stage (P12). (**F**) Bar chart showing comparison of axon numbers innervating the LALc in control (grey) and SmnΔ7 mice (white) across time, from P1 to P12. By P1 there was already a significant loss of axons in this selectively vulnerable muscle, which persisted until end stage. (**G**-**L**) Micrographs and bar charts showing axon number across time in control and SmnΔ7 mice in other, differentially vulnerable, cranial muscles. (**G-H**) The vulnerable AS muscle had a profound loss of axons by P12, with a drop from 24 ± 5 axons in control to 4 ± 1 in SmnΔ7 mice. A significant loss of axons was first seen at P3. (**I-J**) The moderately vulnerable AAL muscle also had significant axon loss by P3, which persisted to end stage. (**K-L**) By P12 even the resilient LALr muscle had significantly fewer axons in the SmnΔ7 muscles compared to controls. Control data is shown in grey and SmnΔ7 in white throughout. *N* = 3–14 mice per time point. Two-way ANOVA with Tukey’s (**B**) or Sidak’s (**D**-**L**) multiple comparisons, * *P* ≤ 0.05, ** *P* ≤ 0.01, *** *P* ≤ 0.001, **** *P* ≤ 0.0001. Scale bar = 50 μm (**A**, **E**, **G**, **I**, **K**) and 100 μm (**C**)

To address how the temporal breakdown of the motor unit manifests, we profiled denervation, endplate loss and axon number in the highly vulnerable LALc cranial muscle in the SmnΔ7 mouse model of SMA from P1 to P12 (end-stage in our hands) (Fig. 2A-F).

The percentage of fully occupied endplates was significantly decreased from P5 and progressively declined to P12, with a corresponding increase in the percentage of partially or vacant endplates (Fig. 2A and B). Quantification of total endplate number revealed a significant reduction at P12 only (Fig. 2C and D). This suggests that endplate loss occurs after structural denervation and emphasises that quantification of innervation status alone underestimates true denervation. Quantification of axon number revealed a 39% loss of motor axons in the intramuscular bundle innervating the vulnerable LALc muscle at P1 (significantly preceding structural denervation and endplate loss) (Fig. 2E and F). This is followed by progressive loss of motor axons until the P12 endpoint.

Quantification of axon number in the AS and AAL muscles in SmnΔ7 mice revealed numbers which were similar to control littermates at P1. However, by P3, both these muscles also had significantly fewer axons, with a 45% loss in the AS (Fig. 2G and H) and 42% loss in the AAL (Fig. 2I and J). The comparatively resilient LALr muscle retained normal numbers of axons at P5, but by P12 displayed a loss of 35% of axons compared to controls (Fig. 2K and L). This work confirms that axon loss occurs in all muscles, but the timing of axonal loss varies between muscles. Together, these findings reveal that axonal loss in SMA is an early event that unites all muscles and precedes both denervation and endplate loss.

### Axonal maturation is largely conserved across differentially vulnerable muscles in SmnΔ7 mice

**Fig. 3:**
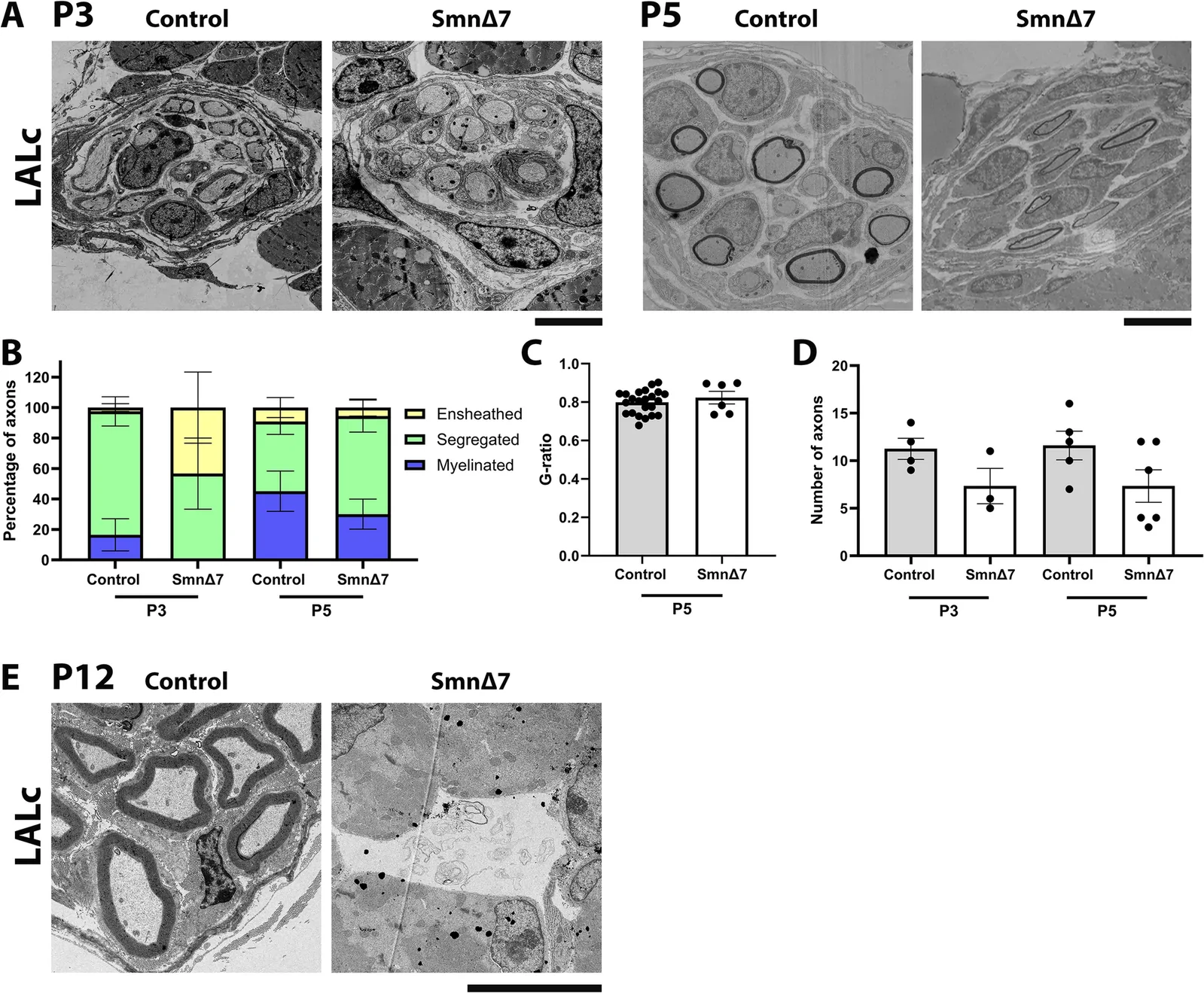
Axonal maturation is largely conserved in the vulnerable LALc muscle, prior to total collapse of axon morphology by P12. (**A**) Electron micrographs showing cross sections of intramuscular axon bundles innervating LALc muscles in control and SmnΔ7 mice at P3 and P5. (**B**) Percentage of axons of different morphological types in LALc intramuscular axon bundles at P3 and P5 (*n* = 3–6 mice per time point. Two-way ANOVA with Tukey’s multiple comparisons test). (**C**) G-ratio of myelinated axons in LALc control and SmnΔ7 mice at P5 (*n* = 23/6 axons from 5/4 mice, Unpaired t-test). (**D**) Total number of axons per axon bundle in LALc muscles (*n* = 3–6 mice. One-way ANOVA with Tukey’s multiple comparisons test). (**E**) Electron micrographs showing cross sections of intramuscular axon bundles innervating LALc muscles in control and SmnΔ7 mice at P12. All Scale bars = 5 μm

**Fig. 4:**
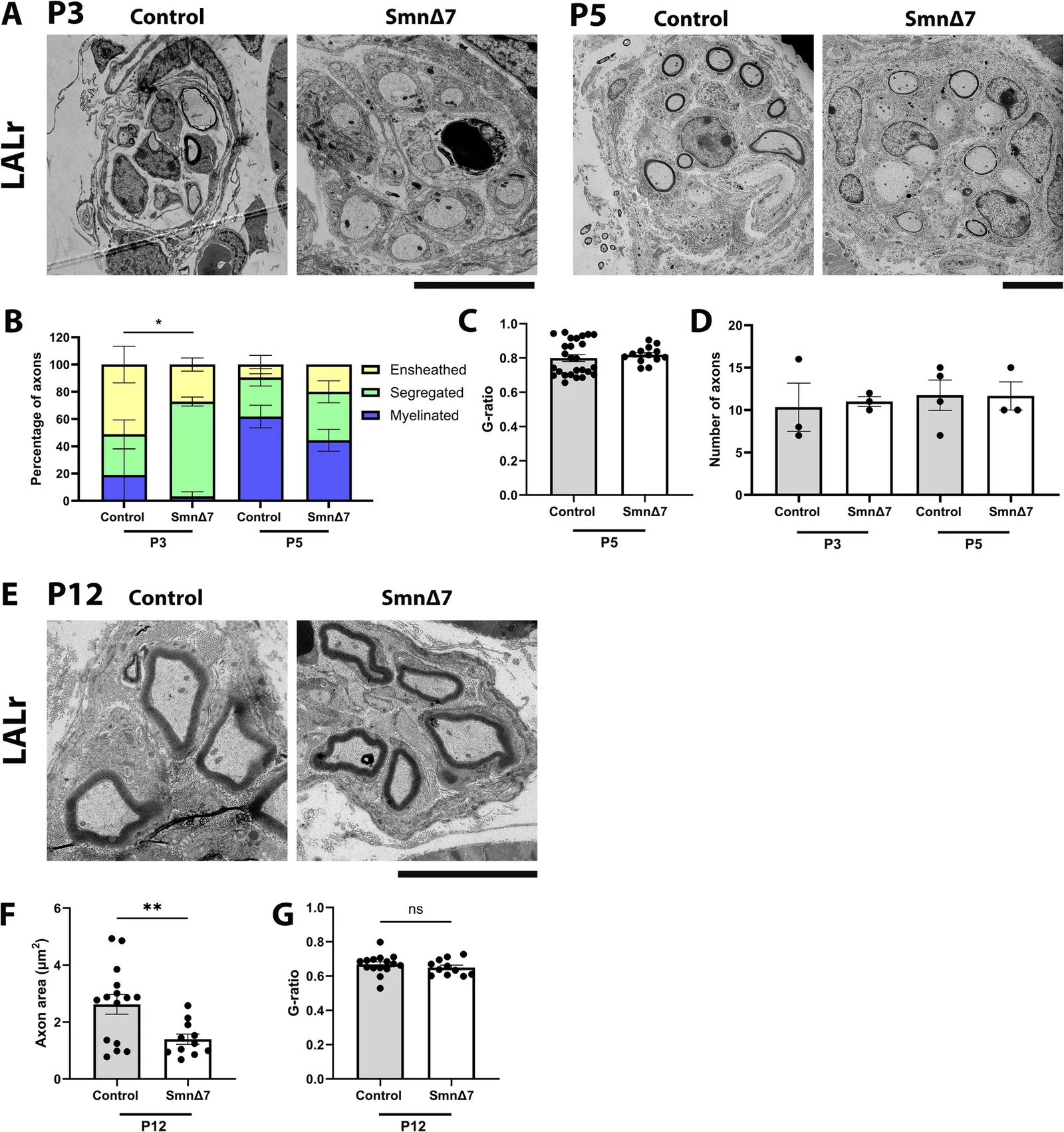
Axonal maturation is largely conserved in the resilient LALr muscle in SmnΔ7 mice. (**A**) Electron micrographs showing cross sections of intramuscular axon bundles innervating LALr muscles in control and SmnΔ7 mice at P3 and P5. (**B**) Percentage of axons of different morphological types in LALr intramuscular axon bundles at P3 and P5 (*n* = 3–4 mice per time point. Two-way ANOVA with Tukey’s multiple comparisons test, * *P* ≤ 0.05 for segregated axons). (**C**) G-ratio of myelinated axons in LALr control and SmnΔ7 mice at P5 (*n* = 26/13 axons from 4/3 mice, Unpaired t-test). (**D**) Total number of axons per axon bundle in LALr muscles (*n* = 3–4 mice. One-way ANOVA with Tukey’s multiple comparisons test). (**E**) Electron micrographs showing cross sections of intramuscular axon bundles innervating LALr muscles in control and SmnΔ7 mice at P12. (**F**) Myelinated axon area in LALr muscles at P12 (*n* = 15/11 axons from 3/2 mice, Unpaired t-test, ** *P* ≤ 0.01). (**G**) G-ratio of myelinated axons in LALr control and SmnΔ7 mice at P12 (*n* = 15/11 axons from 3/2 mice, Unpaired t-test). All scale bars = 5 μm

Previous work has described a population of immature abutting axons in the ventral roots of SmnΔ7 mice which are selectively vulnerable to perinatal degeneration [25]. To investigate whether delays in axonal maturation could contribute to postnatal axon degeneration, we visualised intramuscular axon bundles in LALc and LALr muscles across the lifespan of SmnΔ7 mice (Figs. 3 and 4). Initial inspection of intramuscular axon bundles allowed us to visualise profiles of axons and their associated Schwann cells. Axon numbers in control samples correlated well with fluorescently stained images; in the LALc at P5 11.6 ± 3.4 axons were counted using electron microscopy (Fig. 3D), compared to 10.1 ± 1.2 using immunofluorescence, in the LALr 11.8 ± 3.6 axons were counted using electron microscopy (Fig. 4D), compared to 9.0 ± 2 using immunofluorescence (cf. Fig. 2). This gave confidence that the appropriate intramuscular bundle was being analysed.

To quantify axonal maturation, axons were assigned to one of three groups based on their appearance and relationship to surrounding Schwann cells: myelinated - axons surrounded by myelin; segregated - unmyelinated axons with a 1:1 relationship with a Schwann cell; ensheathed - unmyelinated axons surrounded by Schwann cell which also surrounded other axons. Small-diameter abutting axons were also observed within intramuscular bundles. However, these were excluded from further analysis due to the inability to definitively distinguish between autonomic and motor fibres.

At P3, control LALc muscles primarily contained segregated or myelinated axons. In contrast, SmnΔ7 muscles exhibited primarily segregated or ensheathed axons, indicating a shift towards more immature morphologies, though this did not reach statistical significance (Fig. 3A and B). By P5, the maturation profile in SmnΔ7 muscles was largely indistinguishable from controls (Fig. 3A and B). Those axons which were myelinated exhibited G-ratios similar to controls (Fig. 3C), suggesting that the myelination process itself remains functionally intact once initiated.

Interestingly, total axon numbers were not significantly reduced in SmnΔ7 muscles at either P3 or P5 (Fig. 3D). This is sharp contrast to our analysis of fluorescently labelled axon bundles, where we observed a consistent and significant reduction at both P3 and P5 (cf. Fig. 2). In addition, we did not observe any evidence of classical axon degeneration in SmnΔ7 muscles at either P3 or P5 (Fig. 3A), such as watery axoplasm due to loss of intermediate filaments, accumulation/swelling of mitochondria or degenerating myelin profiles. However, by P12, there had been a total collapse of motor axon morphology in LALc SmnΔ7 muscles, and we were unable to identify any remaining axon bundles, consistent with the vulnerable profile of this muscle (Fig. 3E). Collectively this data shows that, despite modest delays in axonal maturation, segregation and myelination of vulnerable axons is grossly normal prior to their subsequent degeneration.

In the LALc muscle at P3, we estimate around 50% of vulnerable axons have already been lost. We therefore also examined axonal profiles in the LALr muscle at P3 and P5, which are both prior to the subsequent axon loss which occurs by P12 (cf. Fig. 2). Quantification of axons at P3 revealed a modest but significant increase in segregated axons (Fig. 4A and B), but by P5 control and SmnΔ7 muscles had grossly indistinguishable maturation profiles (Fig. 4A and B). Once again, G-ratio was conserved, indicating normal myelin thickness (Fig. 4C). Axon counts in the LALr revealed numbers which were consistent with fluorescent imaging (Fig. 4D). At end-stage (P12) the LALr axons in SmnΔ7 muscles were significantly smaller than those in controls (Fig. 4E and F), but the G-ratio was unaffected (Fig. 4G).

These findings suggest that while there are potential modest delays in axonal maturation, axonal maturation and myelination is grossly intact at time points coinciding with or prior to active degeneration.

### Degenerating axons can be visualised in vulnerable intramuscular axon bundles in SmnΔ7 mice

From the data presented above, we were intrigued by both the disconnect in axonal numbers quantified by fluorescent versus electron microscopy (the significant loss of axons quantified in the LALc at P3 and P5 using NF-M staining but no loss of axons as assessed by EM at P3 and P5 in the same muscle) and by the lack of traditional evidence of axonal degeneration (absence of myelin breakdown, cytoskeletal and mitochondrial abnormalities). Consistent with our observations during EM, during immunohistochemical analysis the absence of fragmented axons or retraction bulbs within the axon bundle was notable. Axon loss was inferred as numbers decreased, but axons in a degenerative state could not be resolved.

To ensure comprehensive labelling of the axonal cytoskeleton, we initially co-stained LALc muscles at P5 with antibodies against neurofilament light chain (NF-L) and neurofilament medium chain (NF-M) (Supplementary Fig. 2). This revealed complete colocalisation of NF-L with NF-M, confirming that our labelling strategy was not confounded by differences in intermediate filament expression.

**Fig. 5:**
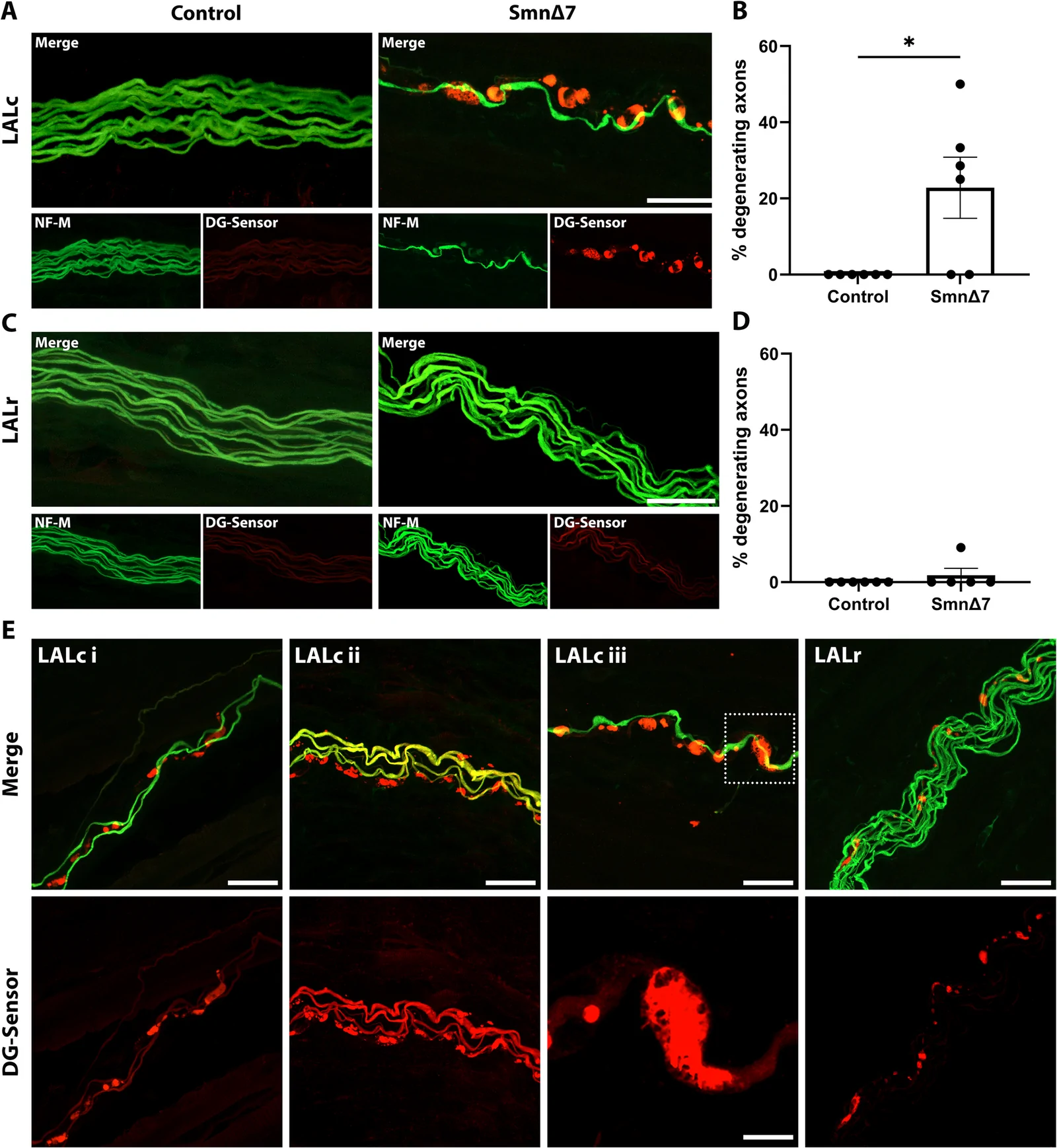
Degenerating axons can be visualised in vulnerable intramuscular axon bundles in SmnΔ7 mice. (**A**) Confocal micrographs showing axons innervating the LALc muscle in control and SmnΔ7 mice at P5. Axons have been labelled with markers for NF-M (green) and for a short region in the centre of the neurofilament light domain, which is only exposed in degenerating or degenerated axons (DG-Sensor, red). DG-Sensor staining, indicating degenerating axons, is seen in the SmnΔ7 LALc, but not in controls. (**B**) Quantification of the percentage of axons in intramuscular axon bundles expressing DG-Sensor in control and SmnΔ7 LALc muscles, showing a significant increase in SmnΔ7 axons displaying DG-Sensor staining (*n* = 6 muscles from 6 mice, Unpaired t-test, *P ≤ 0.05). (**C**) Confocal micrographs showing axons innervating the relatively resilient LALr muscle in control and SmnΔ7 mice at P5. DG-Sensor is not seen in the SmnΔ7 LALr muscles. (**D**) Quantification of the percentage of axons in intramuscular axon bundles expressing DG-Sensor in control and SmnΔ7 LALr muscles showing no significant increase in DG-Sensor staining in SmnΔ7 muscles (*n* = 6 muscles from 6 mice). (**E**) Panel of confocal micrographs showing examples of DG-Sensor staining in SmnΔ7 LALc muscles (LALci-iii), and the only evidence of degenerating axons in the LALr found at this time point (P5, LALr). Scale bars = 20 μm (in magnification of Fig. E LALc iii, scale bar = 5 μm)

To gain further insight into the molecular mechanics of axonal breakdown, we co-immunostained LALc and LALr cranial muscles at P5 using antibodies against NF-M and NF-L DG-Sensor, a novel antibody that recognises a short region in the centre of a neurofilament light domain, which is only exposed in degenerating or degenerated axons [28]. As expected, NF-M positive axons were present in both muscle types across genotypes, though the SmnΔ7 LALc exhibited a reduced number of axons (Fig. 5A). DG-Sensor staining revealed axons which were swollen and beaded, suggesting cytoskeletal breakdown and accumulation of debris (Fig. 5A and E). These degenerating profiles, which expressed little to no NF-M, were found in axon bundles in the SmnΔ7 LALc muscle, but were absent in both control littermates and the more resilient LALr muscle (Fig. 5A-D). Quantification of the percentage of axons expressing DG-Sensor in an axon bundle confirmed a significant increase in the percentage of SmnΔ7 LALc intramuscular axons expressing the DG-Sensor epitope compared to controls (Fig. 5B).

Examination of six SmnΔ7 LALr muscles revealed only a single DG-Sensor positive axon (Fig. 5D and E LALr). This supports the hypothesis that axons in the LALr will eventually degenerate, but on a delayed timeline compared to those in the LALc.

A second DG-Sensor profile was observed but not quantified, and was most prevalent in the SmnΔ7 LALc muscle; a subset of axons were labelled with both NF-M and high levels of DG-Sensor, without evidence of axonal blebbing. This suggests that these axons were in a transitional state of degeneration where the axonal cytoskeleton is still present, but damaged enough to expose epitopes which are usually hidden (Fig. 5E. LALc ii).

The use of DG-Sensor antibodies therefore provides clear evidence of axonal degeneration, even in axons which have become negative for neurofilament medium chain during their degeneration.

### Early SMN-C8 treatment preserves axons in select muscles in SmnΔ7 mice

We have previously shown a persistent loss of axons following SMN up-regulation in vulnerable muscles [26]. However, it is unclear whether this is due to axonal loss prior to and/or after treatment, and to what extent axon regeneration can occur following SMN up-regulation. To address these questions, we treated SmnΔ7 mice with an analogue of the approved treatment Risdiplam (SMN-C8). Mice were treated with a daily intraperitoneal injection of SMN-C8 at a dose of 1 mg/kg. Treatment was initiated at either P2 or 3, and continued until postnatal day 12, (end stage for SmnΔ7 mice which had not received SMN-upregulating therapy).

**Fig. 6:**
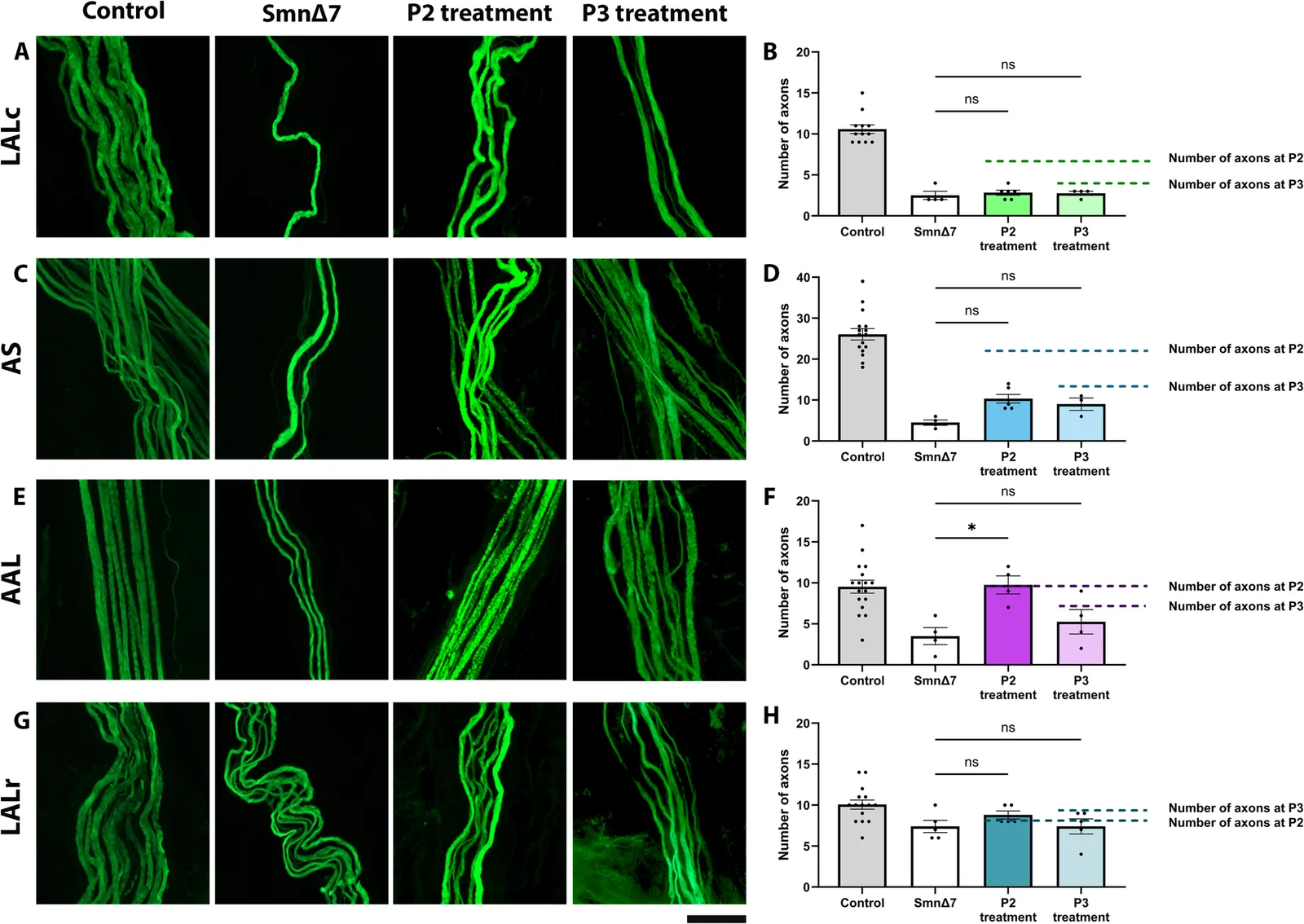
Early SMN-C8 treatment preserves axons in select muscles in SmnΔ7 mice (**A**) Confocal micrographs showing axons innervating the LALc muscle at P12 with and without SMN-C8 treatment. Images show control and untreated SmnΔ7 muscles, and muscles treated with daily SMN-C8 from either P2 or P3. (**B**) Quantification of axon numbers in LALc muscles at P12 with and without SMN-C8 treatment. Dotted lines show the average number of axons in these muscles at the point when treatment was initiated (7 axons at P2 and 4.5 axons at P3, based on data presented in Fig. 2). (**C-D**) micrographs and quantification of axons in the AS muscle with daily SMN-C8 treatment. As with the LALc, treatment was not sufficient to significantly preserve axon numbers. (**E-F**) micrographs and quantification of axons in the AAL muscle with daily SMN-C8 treatment. Treatment from P2 was sufficient to significantly preserve axon numbers. (**G-H**) micrographs and quantification of axons in the LALr muscle with daily SMN-C8 treatment. Control data is shown in grey, untreated SmnΔ7 in white and treated SmnΔ7 in colour throughout. *n =* 3–17 mice. One-way ANOVA with Tukey’s multiple comparisons, * *P* ≤ 0.05, ** *P* ≤ 0.01. Scale bar = 50 μm

In the LALc muscle, where there are already significantly fewer axons by P1, a significant loss of axons was evident at P12 compared to treated control mice, and there was no significant difference compared to untreated SmnΔ7 mice (Fig. 6A and B). There was also no significant difference between the groups treated on day 2 or day 3.

In the AS and AAL muscles, where axon loss commences between 2 and 3 days (cf. Fig. 2), we similarly saw a significant loss of axons compared to control. However, we found that only the AAL muscle responded significantly to early intervention; mice treated with SMN-C8 at P2, prior to significant loss of axons, retained significantly more axons in the AAL muscles than untreated SmnΔ7 mice (Fig. 6E to F). Conversely, P2 treatment did not lead to a significant increase in axon numbers in the AS muscle (Fig. 6C to D). When initiation of treatment was delayed by 24 h to P3, there was no significant difference in axon number in SMN-C8 treated mice and untreated SmnΔ7 pups in either the AS or AAL muscles (Fig. 6C to F).

As the resilient LALr muscle has only mild axon loss, which begins at the end stage time point, there was no significant decrease in untreated mice compared to controls and therefore no significant improvement in axon number in this muscle following P2 or P3 treatment. (Fig. 6G and H).

Collectively this data suggests that treatment with SMN-upregulating SMN-C8 can, at most, preserve axon numbers at the time of treatment but does not measurably reverse damage which has already occurred. It also highlights that there are critical, distinct vulnerabilities even among muscles with similar timelines of degeneration where small delays in treatments can have impactful long-term consequences. There is a wealth of evidence to show that earlier treatment leads to better outcomes in patients receiving SMN-upregulating therapies [30–32], and this finding suggests that this could be in part due to a loss of motor axons which are not recovered by current treatment regimes.

## Discussion

The data presented here highlight the importance of intramuscular axons in the progression of SMA. Notably, loss of motor axons precedes detectable denervation and was common to all muscles examined. However, there was a differential time course of axonal loss which drives relative vulnerability. We have shown that postnatal maturation and myelination of axons is largely conserved prior to their subsequent loss, and that there is an absence of classical degenerative axonal profiles. We further show that degenerating axons can be visualised using antibodies targeting degenerating neurofilaments, and that axons become negative for traditional neurofilament antibodies as they degenerate. Importantly, SMN-C8 treatment at best preserved axons only when administered before degeneration began, with no evidence of an increase in axon numbers following treatment. This work highlights the importance of early therapeutic intervention and the potential for Smn independent therapies to further protect and regenerate vulnerable axons.

### Divergent signatures of motor unit pathology are united by axonal loss

Vulnerability of muscles to motor neuron diseases has previously been assigned based solely on loss of innervation at the NMJ, which is an early and important event, signifying loss of function of the muscle [13]. Here we have evaluated a range of morphological parameters of the distal motor unit and reveal at least three distinct profiles amongst the four muscles examined. The LALc and AS muscles had remarkably similar profiles; high denervation levels were accompanied by a severe loss of endplates and axons and pre-synaptic swelling. By comparison, the AAL displayed remarkable plasticity wherein a similarly severe loss of axons was compensated for by an increase in motor unit size which protected the muscle from denervation and preserved endplate numbers. In contrast, the LALr muscle displayed relative resilience; the most notable feature in this muscle was an increase in pre-synaptic swelling, but there was no obvious denervation and only a small decrease in endplate number and axon number. These three divergent profiles amongst four anatomically grouped muscles which share common innervation from the facial motor nucleus raise interesting questions about the mechanisms which mediate motor neuron pathology and resilience in SMA and how they are differentially regulated between muscles. However, these divergent phenotypes also highlight the importance of considering multiple muscles when profiling motor unit pathology and response to treatment. Since profiling multiple muscles in a clinical scenario is not always feasible, pre-clinical models will play a key role in describing differential phenotypes and in elucidating defining characteristics which can inform clinical studies and facilitate their design and interpretation.

Despite the divergence in profile, all muscles examined were united by a loss of motor axons. It is worth noting that our axonal quantification was restricted to the cranial muscles. Evaluating early motor axon loss in respiratory and axial muscles such as the diaphragm or trapezius would provide a broader understanding of muscle vulnerability, but the dense and complex innervation patterns in larger muscles prevent the precise isolation and total quantification of innervating axons. In contrast, the cranial muscles are innervated by highly consistent and identifiable intramuscular axon bundles, in which we found that axon loss is an early pathological event, preceding other detectable signs of neuromuscular degeneration and present in muscles previously considered resistant [29]. In the most severely affected muscles, a significant reduction in axon number was already evident by P1. Loss of innervation at the NMJ was not seen until day five, suggesting that remaining axons may undergo compensatory sprouting, temporarily preserving NMJ integrity despite the loss of neighboring axons, or that high levels of poly-innervation at birth may initially mask the loss of neighbouring axons. This plasticity underscores the inherent resilience of the neuromuscular system. It also highlights the mechanism by which high levels of degeneration can occur in clinically silent phases of the disease. Whilst compensatory sprouting and motor unit enlargement can act to preserve motor function in the short term, the long-term fate of enlarged motor units is not known. Evidence from the post-polio syndrome field suggests that the heightened physiological stress upon enlarged motor units may limit their longevity and lead to motor unit loss and associated weakness later in life [33]. As such, monitoring motor unit sizes in people with SMA, regardless of how early they were treated, remains critical and efforts to understand and mitigate the stresses put upon enlarged motor units are warranted.

Together, these findings redefine the timeline of neuromuscular pathology, positioning axonal degeneration as a primary and early event, and emphasising the importance of early detection and intervention strategies aimed at preserving axonal integrity to maintain motor unit size before NMJ disruption becomes apparent.

### Axonal maturation and myelination are largely preserved prior to rapid degeneration

Our ultrastructural analysis provides evidence that the selective vulnerability and degeneration of intramuscular motor axons in SMA is not as a direct consequence of a failure to mature. Although we observed a modest delay in maturation at P3, by P5 maturation was indistinguishable from wild type controls. Myelin sheaths, where present, exhibited normal thickness relative to axon diameter. This suggests that the molecular machinery required for radial sorting and early ensheathment is functional in the postnatal SMA motor unit.

In their study of prenatal motor axon development in SMA ventral roots, Kong et al. [25] showed that development is impaired prenatally, leading to reduced radial growth and ensheathment, and is followed shortly after birth by rapid degeneration of immature axons [25]. Our data confirms the presence of an early, potentially prenatal, deficit, evidenced by the 39% loss of axons in the LALc by P1. Kong et al. also reported that remaining proximal axons acquired more mature morphologies, which is in agreement with our finding that SmnΔ7 intramuscular bundles maturation profiles and myelin thickness are grossly normal. This suggests that while perinatal axonal loss targets developmentally immature axons, subsequent postnatal degeneration appears to occur independently of structural maturity. Although postnatal axons successfully reach key maturational milestones, this transition into a mature state does not protect them from degeneration. This underscores the importance of combining SMN-upregulation with neuroprotective strategies that stabilise the axon during this highly dynamic and vulnerable phase.

### Visualising degenerating axons

Axon degeneration can occur in response to disease, injury or chemical insult and has been intensely studied. The morphology of traditional Wallerian axon degeneration has been well characterised, involving axonal blebbing and fragmentation, cytoskeletal breakdown, mitochondrial swelling and unravelling of the myelin sheath [34]. Die-back pathology has been described in SMA, typically involving a distal to proximal withdrawal of a retraction bulb whilst cellular organelles generally remain intact [14]. We therefore found it notable that, when studying both fluorescently stained axons bundles and cross-sectional ultrastructure, we did not observe any of these hallmarks within the intramuscular axon bundle. Here axon loss is inferred based on reduced axon numbers, but we did not detect morphological characteristics of axon degeneration.

Use of the DG-Sensor antibody provides the first direct morphological evidence of axonal degeneration within vulnerable intramuscular motor axon bundles in SMA. The use of DG-Sensor, which binds to an epitope exposed only during axonal degeneration, allowed us to overcome this limitation and directly visualise degenerating axons [28]. DG-Sensor staining revealed clear evidence of axonal degeneration specifically in the selectively vulnerable LALc muscle of SMA mice. In contrast, the more resilient LALr muscle and control littermates showed no such staining, with only a single DG-Sensor positive axon seen across all LALr muscles examined. As DG-Sensor positivity represents a transient stage of active breakdown, its rare presence in a single LALr sample at P5 suggests that while these resilient axons may eventually degenerate, they possess a significantly higher threshold of resistance compared to the rapidly degenerating LALc.

Interestingly, some axons that were positive for NF-M also exhibited strong DG-Sensor staining, suggesting they were in a transitional state of degeneration. These axons likely retain elements of their cytoskeletal structure while undergoing pathological remodeling, exposing normally hidden epitopes. Additionally, we observed axons that lacked NF-M staining altogether but appeared swollen and beaded, consistent with advanced cytoskeletal breakdown and debris accumulation. These morphological features further validate DG-Sensor as a tool for capturing a spectrum of degenerative states. They also highlight that as axons degenerate, they become negative for traditional markers such as NF-M. Perhaps this may, in part, explain the difficulty in identifying degenerating motor neurons in SMA, as traditional markers such as choline acetyl transferase or HB9 may be lost as the cell degenerates.

The ability to directly visualise degenerating axons provides a novel way to map axonal pathology in SMA and could provide a marker for early degenerative events in susceptible motor axons. This could prove important in evaluating therapeutic interventions aimed at preserving neuromuscular connectivity. Further studies are now required to assess axonal degeneration across time and in other muscle groups, and to assess whether DG-Sensor can detect potentially reversible stages of pathology.

### The importance of treatment timing and clinical implications

We found that administration of SMN-C8 prior to significant axonal degeneration significantly preserved intramuscular axons compared to untreated pups. This protective effect was entirely lost when treatment was delayed by just 24 h, underscoring the narrow therapeutic window in which SMN-C8 is effective at rescuing axon number. These findings indicate that SMN-C8 is capable of preserving axonal integrity but not reversing existing damage. We note that our study did not track individual axons longitudinally or evaluate longer post-treatment survival intervals. Whilst there is no evidence for axon regeneration under the specific conditions tested here, future studies with extended timelines may provide further nuance.

Our analysis of differentially vulnerable muscles highlights that all muscles follow their own time course and that the critical therapeutic window will depend on the muscle which is being assessed. This has important implications for clinical studies tracking pre- and post-treatment electrophysiological deficits, where typically only one muscle is evaluated. Whilst this is entirely understandable with regards to feasibility, it should be noted that it is a limitation when the information about one muscle is used as a biomarker to explain or predict motor function throughout the body.

These observed early and irreversible loss of motor axons offer an explanation for clinical observations in human SMA patients; pre-symptomatic infants treated shortly after birth in the NURTURE [11] and RAINBOWFISH [12] trials achieved developmental milestones, such as independent walking, that are almost never reached by infants treated after the onset of symptoms (e.g., in the ENDEAR [3] or FIREFISH [35] trials). Our data suggests that this may be due to earlier treatment leading to the preservation of a greater percentage of motor axons and provides a cellular explanation for the clinical ‘time is motor neuron’ paradigm. This reinforces the critical importance of newborn screening and immediate therapeutic intervention to catch muscles within their respective windows of vulnerability, as lost axons represent a permanent functional deficit that current SMN-targeted therapies cannot rescue.

While our findings provide insights into early cellular pathology, we must acknowledge the inherent limitations of the model used. The SmnΔ7 mouse model is a severe neonatal SMA model with an early phenotype with a highly compressed disease trajectory. Consequently, the pathological mechanisms observed within the cranial musculature of this mouse model may not perfectly recapitulate the degeneration seen in human patients, especially those with milder, late-onset SMA, and care should be taken when translating these findings to the broader clinical presentation of late-onset disease.

As SMN-upregulation alone is insufficient to reverse the loss of axons in SMA, future treatment strategies should combine SMN-upregulating therapies with those which aim to support and regenerate motor axons, in an attempt to reinnervate muscles.

## Conclusions

In summary, we have shown that intramuscular axons are an early pathological target in SMA, with axon loss preceding NMJ denervation. This finding suggests that assessment of vulnerability of muscles should move beyond NMJ innervation alone to include axonal and motor unit pathology. Importantly, SMN-C8 treatment preserved axons only when administered before degeneration began, emphasising the necessity of early intervention. The use of DG-Sensor to visualise degenerating axons offers a powerful tool for mapping early pathology and evaluating therapeutic efficacy in SMA.

Taken together this work further strengthens the case for early treatment of SMA, including more prevalence of newborn screening and the earliest possible administration of SMN-upregulating therapies. It shows that axons are a key pathological target in SMA and should be a focus for future combinatorial therapeutics to improve long term motor neuron health and function to provide the best possible outcomes for patients.

## Supplementary Information

Below is the link to the electronic supplementary material.

Supplementary Material 1

Supplementary Material 2

## Data Availability

The datasets used and/or analysed during the current study available from the corresponding author on reasonable request.

## Abbreviations

AAL: Abductor auris longus
AChR: Post-synaptic acetylcholine receptor
AS: Auricularis superior
ASO: Antisense oligonucleotide
BTX: α-bungarotoxin
LALc: Levator auris longus (caudal)
LALr: Levator auris longus (rostral)
NF-L: Neurofilament-light
NF-M: Neurofilament medium
NMJ: Neuromuscular junction
P: Postnatal day
SEM: Scanning electron microscope
SMA: Spinal muscular atrophy
STEM: Scanning transmission electron microscope
SV2: Synaptic vesicle proteins
